# Evisceration of Abdominal Contents Through a Posterior Vaginal Defect: A Case Report

**DOI:** 10.1155/crog/9915866

**Published:** 2025-09-22

**Authors:** Emily Nguyen, Jennifer Ferraro, Valerie Galvan-Turner, Iheanacho Emeruwa

**Affiliations:** Division of Obstetrics and Gynecology, Riverside Community Hospital, Riverside, California, USA

## Abstract

Vaginal evisceration is a very rare complication after a hysterectomy, and it can have extremely dire health consequences. We present a case of a 65-year-old female with a past medical history significant for obesity, pelvic organ prolapse, and rheumatoid arthritis—treated with chronic oral steroids—who presented with transvaginal small bowel evisceration more than 20 years after a total vaginal hysterectomy. The patient underwent emergent exploratory laparotomy with reduction of the bowel and reclosure of her vaginal cuff. The patient's postoperative course was complicated by bowel ischemia and subsequent bowel resection. We report this case to bring awareness to risk factors associated with vaginal evisceration; early diagnosis of this dangerous hysterectomy sequelae is vital to prevent life-threatening bowel ischemia.

## 1. Introduction

Transvaginal small bowel evisceration is a rare event that typically requires emergent surgical intervention; the rate of occurrence can vary depending on the type of surgical procedure but is overall estimated to be between 0.032% and 1.2% [1, 2]. While there are various etiologies of this condition reported in the literature, the majority of transvaginal small bowel evisceration cases occur in postmenopausal women after hysterectomy [1, 2]. The first documented case of vaginal evisceration was described in 1864 by Hyernaux; a patient had her abdominal contents prolapse through the anterior wall of the proximal vagina [3, 4]. Over 120 reports have been noted in the literature since this first case, though many cases may go unreported [5]. Most cases reported occur in postmenopausal women with subsequent vaginal cuff dehiscence after hysterectomy [6, 7]. The management and surgical approach typically depend on the viability of the bowel, the size of the vaginal defect, and the ability to replace the bowel back into the abdominal cavity using the existing defect. Within the literature, laparoscopic, open abdominal, transvaginal, or a combination of those approaches has been described in the surgical management of this issue [5, 8, 9]. Due to its rare nature and the infrequency in which evisceration of abdominal contents through vaginal defects is encountered, medical personnel can fail to diagnose and act on this issue early. However, hasty detection and prompt surgical management are necessary to avoid catastrophic results, including bowel ischemia and death. The goal of this case report is to provide attention to this rare issue to highlight the importance of early detection and management, as well as re-emphasize additional risk factors that increase the risk of evisceration.

## 2. Case Presentation

A 65-year-old Gravida 6 Para 6 woman presented to the emergency department (ED) with pelvic pain. The patient's past medical history was significant for morbid obesity, Type II diabetes mellitus, hypertension, rheumatoid arthritis—on chronic prednisone therapy, pelvic organ prolapses, and chronic constipation. Her surgical history included multiple abdominal surgeries, including a total vaginal hysterectomy and a failed bladder sling 20 years prior. Her pelvic pain was initially attributed to presumed pelvic organ prolapse, as the patient described feeling something bulge out of the vagina earlier in the day while walking. She initially assumed it was her bladder, given her history of hysterectomy and bladder sling, so she attempted to reduce it as she would normally. However, she developed worsening abdominal pain and nausea, prompting her visit to the ED. At the time of her ED visit, she endorsed pelvic pain that radiated to her back, nausea, and multiple episodes of emesis. The initial evaluating ED physician suspected that her symptoms were consistent with pain from pelvic organ prolapse.

**Figure 1:**
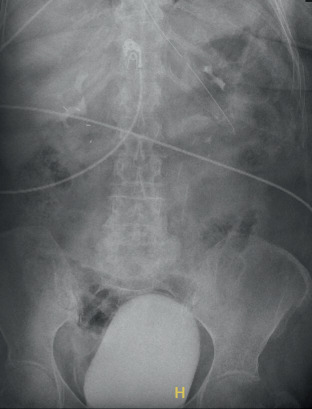
Abdominal x-ray.

**Figure 2:**
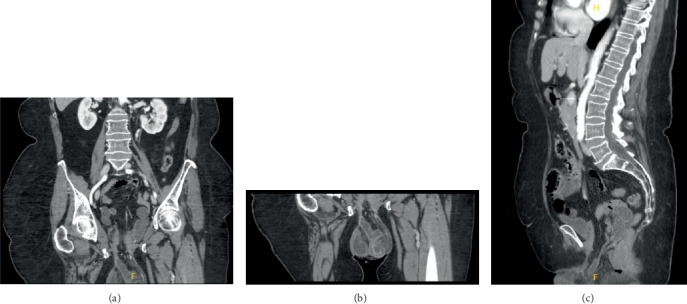
(a) Coronal CT imaging of the abdomen and pelvis. (b) Coronal CT imaging of the pelvis. (c) Sagittal CT imaging of the abdomen and pelvis.

While in the ED, the patient was in visible distress due to pain. She was found to be afebrile but hypertensive with systolic blood pressures in the 190s. Labs revealed leukocytosis but were otherwise unremarkable. Physical examination was significant for pelvic floor prolapse with dilated “beefy red bowels” noted outside of the vaginal introitus. Abdominal x-ray and CT imaging confirmed vaginal evisceration with signs of strangulation of the small bowel (Figures 1, 2(a), 2(b), and 2(c)). Upon diagnosis of small bowel evisceration and prolapse into the vagina, the patient's care team planned for emergent exploratory laparotomy with replacement of the bowel into the abdomen and reclosure of the vaginal cuff.

## 3. Hospital Course

The patient was taken back for emergent exploratory laparotomy and reduction of small bowel into the abdomen with closure of the vaginal cuff with the gynecology and general surgery team. She was provided ampicillin, gentamicin, and clindamycin preoperatively for broad spectrum and intra-abdominal and pelvic bacteria coverage. During the exploratory laparotomy portion, general surgery performed a reduction of the bowel into the abdominal cavity by providing both external pressure through the vagina and intra-abdominal counter pressure. Once reduced, gynecology then repaired the vaginal defect. Initially, it was presumed that the defect was located at the vaginal cuff given the patient's history of hysterectomy. However, during the procedure, the etiology of the evisceration was found to instead be due to a large perforation of an enterocele in the posterior vaginal wall. The enterocele was then repaired in two layers using purse string sutures and 0-vicryl to occlude the posterior cul-de-sac. This was followed by a posterior vaginal repair also using 0-vicryl and sacrospinous ligament fixation. Additionally, general surgery found mesenteric defects most likely created during the reduction of the bowel into the abdominal cavity. These linear defects were parallel to the bowel arcades created in the mesentery. They performed an indocyanine green angiography that demonstrated viable and well perfused bowel proximal and distal to these mesenteric defects.

Postoperatively, the patient was hypotensive and tachycardic in the postanesthesia care unit despite 3 L of resuscitation fluids. The decision was made to return the patient to the operating room for a repeat exploratory laparotomy to ensure there was no bleeding from the mesenteric rents. Intraoperative findings were significant for indurated small bowel, which was resected due to concerns of ischemia and subsequent sepsis. Bowel resection was performed using impact LigaSure, followed by side-to-side anastomosis using a 60-mm blue load GIA stapler, and the enterotomy was closed with a 60-mm TA stapler. The suture lines were imbricated using 3-0 vicryl sutures, and mesentery defects were reapproximated using 3-0 vicryl. After the second exploratory laparotomy, she was treated in the surgical intensive care unit for presumed septic shock. She was provided stress-dose steroids, hydrocortisone 100 mg, due to concern for adrenal insufficiency; this was eventually tapered to her home dose of prednisone. Postoperative antibiotics were switched to Rocephin and Flagyl. During the remainder of her hospital stay, she exhibited daily improvements in her vital signs and symptoms. Final blood cultures showed no growth, and she was ultimately discharged home in stable condition after a 6-day hospital stay.

## 4. Discussion

The first case of transvaginal evisceration was described in 1864 by Hyernaux; since then, there have been slightly over 120 cases documented [10]. Presenting symptoms of this issue are acute pelvic pain, bleeding, and vaginal discharge [11]. Differential diagnosis of acute abdominal or pelvic pain in postmenopausal women with a history of a hysterectomy should include vaginal evisceration, pelvic organ prolapse, pelvic abscess, pelvic inflammatory disease, and other etiologies such as gastroenteritis, diverticulitis, or appendicitis. It is crucial for there to be immediate examination and action because evisceration may lead to harmful consequences such as ischemia of the bowel, sepsis, and even death [12]. If the color of the bowel suggests necrosis or the patient is unstable—as was seen in this case report, a transabdominal approach should be attempted immediately. However, if the patient is stable without signs of peritonitis, a transvaginal and/or minimally invasive approach can be used for reduction and examination of the bowel [12, 13].

The most common risk factor in premenopausal women typically involves vaginal trauma, usually due to sexual intercourse or foreign bodies [14]. With postmenopausal women, typical risk factors include vaginal surgery, chronic pelvic prolapse, postmenopausal vaginal atrophy, and increased intra-abdominal pressure [3, 15]. Due to a lack of estrogen in the postmenopausal state, the vaginal wall atrophies and thins, and there is weaker pelvic support predisposing the vaginal wall to rupture [15]. Chronic enteroceles can also weaken the vaginal wall more due to chronic stretching, and increased intra-abdominal forces can cause increased mechanical force against a weakened wall, thus leading to rupture [3, 15]. Additionally, long-term steroid therapy can also contribute to spontaneous vaginal rupture. There was one case report that demonstrated spontaneous vaginal evisceration in a patient with iatrogenic Cushing's syndrome [16]. Possible weakening of the vaginal apex was hypothesized to be due to steroid use, which impairs collagen synthesis [16].

Typically, the site of vaginal evisceration is through the site of the vaginal cuff and often is due to cuff dehiscence; about 70% of reported cases occur in postmenopausal women with prior vaginal surgeries [17]. The exact timing of vaginal cuff dehiscence after hysterectomy can be as early as 3 days to as late as 30 years postoperatively [12, 18, 19]. In this case, the patient's evisceration occurred about 20 years after her hysterectomy and went through the posterior fornix. There has been a case report that documented an evisceration through the posterior fornix after coitus [20]. However, for this patient, coitus did not play a role in her evisceration development, but she did digitally attempt to reduce her suspected prolapse, which could have played a role in the development of her perforation. Additionally, while the presence of a weak area due to a poorly healed vaginal cuff after a hysterectomy could be one of the major predisposing factors in this patient, her chronic steroid use and chronic constipation could have also played a significant part in weakening her posterior fornix. There have been a few studies that show the possibility of estrogen study improving the collagen synthesis in vaginal walls; one study in a rat model found high-dose vaginal estrogen could increase vaginal collagen, and another study performed in postmenopausal women found testosterone therapy stimulated collagen synthesis while estrogen–progesterone therapy made a smaller effect on collagen synthesis [21, 22]. However, there are few studies that show whether estrogen therapy or any additional treatment can reverse or reduce the effect of chronic steroid use on vaginal tissue. Future studies should investigate possible treatments and guidelines for the improvement of vaginal integrity for patients on chronic steroids.

We present this case to raise awareness of the different risk factors that can contribute to vaginal evisceration. Additionally, this case emphasizes the importance of knowing presenting symptoms and signs of vaginal evisceration for rapid diagnosis and aggressive treatment to prevent potential bowel ischemia and necrosis.

## Data Availability

Data sharing is not applicable to this article as no datasets were generated or analyzed during the current study.
